# The challenges of trying to increase preventive healthcare for children in general practice: results of a feasibility study

**DOI:** 10.1186/s12875-015-0306-x

**Published:** 2015-08-05

**Authors:** Karyn E. Alexander, Bianca Brijnath, Danielle Mazza

**Affiliations:** Department of General Practice, School of Primary Health Care, Monash University, 270 Ferntree Gully Road, Notting Hill, VIC 3168 Australia

**Keywords:** Pilot study, Complex intervention, General practice, Preventive health care, Child health, Healthy Kids Check

## Abstract

**Background:**

In Australia, general practice, the linchpin for delivery of preventive health care to large segments of the population, provides child-immunisation and preventive health alongside government services. Despite this, less than half of eligible children complete a Healthy Kids Check (HKC), a preschool preventative health assessment available since 2008. Using a rigorous theoretical process, the barriers that affected delivery and reduced general practitioner and practice nurse motivation to provide HKCs, were addressed. The resulting multifaceted intervention, aimed at increasing the proportion of children receiving evidence informed HKCs from general practice, was piloted to inform a future randomised controlled trial.

**Methods:**

The intervention was piloted in a before and after study at three sites located southeast of Melbourne, between February and October 2014. The HKC-intervention involved: 1) Delivery of training modules that motivated reception and clinical staff by delivering key messages about local prevalence rates and the “Core Story of Child Development” 2) Practical advice to prepare clinics for specific HKC-examinations 3) Workflow advice regarding systems that included all staff in the HKC process, and 4) Provision of a “Community Resources Folder” that enabled decision making and referrals. A major component of the intervention incorporated the promotion of structured developmental screening by the practice team using Parents’ Evaluation of Developmental Status.

**Results:**

Twenty of 22 practitioners and practice managers agreed to join the study. Post-training questionnaires showed participants had developed their skills working with young children as a result of the training and all respondents believed they had successfully implemented standardised HKC services. Post intervention proportions of children completing HKCs significantly increased in two of the practices and quality improvements in HKC-processes were recorded across all three sites.

**Conclusion:**

This pilot study confirmed the feasibility of delivering a multi-faceted intervention to increase HKCs from general practice and demonstrated that significant quality improvements could be made. Future studies need to extend the intervention to other states and research the health outcomes of HKCs.

## Background

In Australia, Child and Family Health Nurses (CFHNs) play a key role providing universal, government funded, preventive health to young children, administered at local government level [1]. Despite this, services remain fragmented, the result of divergent policy frameworks established by the eight federated states and territories [1], so that less than 60 % of families complete a pre-school visit [2]. In response to persisting child development and health issues, the Australian government introduced the Healthy Kids Check (HKC) into general practice, in 2008, and made a family tax benefit contingent on receipt of a pre-school check [3]. The HKC is a one-off health assessment, available to children (aged 3.5 to 5 years) completing pre-school vaccinations (with any GP or local government services) (Table 1) [4]. Parents can, therefore, choose not to receive CFHN services and may obtain a HKC from GPs in the private sector. Overall, less than half of eligible children complete a HKC, with wide variability across jurisdictions, from a low of approximately 22 % in Victoria (where CFH services are arguably the most developed [5]), to a high of approximately 66 % in Queensland [6]. Our previous research found that HKC delivery is hampered by a combination of practitioner, environmental and system barriers that combine to reduce motivation [7]. The findings aligned with barriers of ‘insufficient time’ and ‘a lack of community resources’ uncovered in a survey of GPs in Victoria, prior to rollout of the HKC, when delivery of preventive healthcare during ‘sick-child’ consultations was another principal concern [8].

**Table 1:** Mandatory and non-mandatory components of the Healthy Kids Check

Mandatory^a^	Non-mandatory
Height	Discuss eating habits
Weight	Discuss physical activity
Eyesight	Speech and language development
Hearing	Fine motor skills
Oral health	Gross motor skills
Question toilet habits	Behaviour and mood
Note Allergies	Other examinations as necessary

^a^Mandated by Australian government, endorsed by Royal Australian College of General Practitioners [34]

Primary care based interventions focussing on increasing child preventive health services (excluding immunisation) are relatively sparse but have previously tested: the feasibility of an intervention in general practice to prevent child-obesity [9], an oral health intervention [10] and Autism screening amongst CFHNs [11]. In each study the design of the intervention was informed by the barriers identified in previous research [10, 11] or a needs analysis [9] and incorporated training and facilitated referral pathways. In the United States (US), systematic reviews of paediatric primary preventive health interventions have also considered screening for lead poisoning, anaemia and tuberculosis, developmental problems, vision, hearing, and blood pressure. Interventions were generally multifaceted, combined education and training with audit and feedback, included environmental modifications (e.g. clinical decision making aids) and improving office support systems [12, 13]. These interventions suggest potential solutions relevant to the Australian context and to our intervention pilot targeting the HKC.

### Aims

The study aims to test the feasibility of an evidence-based complex intervention designed to increase the proportion of children aged 3.5- 5 years (target age group) receiving HKCs. Secondary aims are to increase the proportion of eligible children having a body mass index (BMI) recorded and increase GP and Practice Nurse (PN) self-efficacy administering HKC services.

## Methods

### Overview

This was a 6 month pilot of a “whole-of-practice” intervention, delivered to three general practices. Quantitative methods, pre- and post-implementation, tested the feasibility and impact of the HKC-intervention on staff beliefs, attitudes and behaviours, and measured components of practice activity. The study was approved by Monash University Human Research Ethics Committee. Written informed consent was obtained from all study participants.

### Setting and participants

Implementation of the intervention took place between February and October 2014 at sites located in the catchment of a single regional primary healthcare organisation (PHO), southeast of Melbourne. Practices served predominantly urban populations in a region experiencing rapid growth with pockets of high social disadvantage [14] and high levels of child developmental vulnerability [15]. Practices were recruited via an advertisement placed in the electronic newsletter of the PHO (158 practices). The project nurse, who also worked to support vaccination services in the catchment, encouraged enrolment into the study. To be eligible, practices had to provide vaccination services to children and propose key personnel- a GP or PN “HKC-Champion” and a practice manager- to drive the intervention and liaise with clinical and office practice staff.

### Design of the intervention

The intervention was constructed following the UK Medical Research Council guidance [16] in an iterative process that applied a behaviour change model [17]. The Behaviour Change Wheel, a tool for systematically designing and evaluating behaviour change interventions, mapped the barriers and facilitators uncovered in our qualitative exploratory work, to specific interventions that could be used to target those factors. Since multiple factors operated to impede the delivery of the HKC, a multi-faceted intervention was required. The intervention included: Education and skills training addressing lack of knowledge about specific components of the HKC and how to communicate sensitive findings to parents (e.g. child-overweight and developmental delay); attending to equipment and space requirements; using a team-based model of care to address time and staffing barriers; and provision of evidence based tools and pre-formulated pathways of care to overcome negative beliefs about the outcomes of HKCs. The intervention was determined following consultation with a stakeholder group.

A significant component of the HKC-intervention was the introduction of the Parents’ Evaluation of Developmental Status (PEDS) questionnaire [18], a tool used to assess child development, validated and widely used by CFHN services in Australia. PEDS was selected because it elicits parent concerns, applies to children of preschool age and is completed, scored and interpreted in 2–5 minutes, making it an ideal choice for general practice.

The HKC-intervention was divided into four areas of content:

A. *Three training modules* that opened with “The Core Story of Child Development” [19] and information about local prevalence rates of child health problems [15] aimed at motivating participants. In addition:
  1. Reception staff and the practice manager- received advice about appointment systems and a business model, and role-played ‘what to say when handing PEDS to the parent’.
  2. “HKC - Champions” were trained how to score and incorporate PEDS into the HKC, and how to perform each of the mandatory HKC components
  3. GPs and PNs were trained on the importance of parent concerns, correct measurement techniques and how to interpret PEDS scores and determine the next step.
B. *Practical advice* that began with ‘Equipment and process inventories’ to record how prepared clinics were for HKCs. Advice directed clinics towards sourcing equipment and problem solving to enable HKC completion. A PEDS pack, containing 50 questionnaires, score and interpretation forms, was supplied to each practice.
C. *Systems advice* established how to set up recall and reminder systems, schedule HKC appointments and maintain supplies and workflow using PEDS.
D. *An electronic ‘Community Resources Folder’* that contained the contact details of local paediatric, early intervention and community services, parent tip-sheets, websites and a number of freely available “secondary” developmental screens (e.g. the Pediatric Symptom Checklist [20]). Folders were installed onto practitioners’ computerised desktops to increase accessibility.

### Procedure

Six visits were planned for each practice, five within the first 3-month ‘active intervention’ phase. The project nurse obtained consent, delivered questionnaires and the education and training modules (3 visits), assisted with equipment and process inventories (Table 2), extracted data (3 visits) and offered advice. She was supported by one of the researchers (KA), a general practitioner with expertise in preventive health of preschool children, whose role was to deliver peer-group clinical training (module 2).

**Table 2:** Inventory of Practice Equipment and Processes used for HKCs

Quality indicator	Description
Office systems	Uses a recall or reminder system to invite or identify eligible children
	Has a process in place to deliver PEDS^a^ to parent in waiting room
	Has a list of referral sources (e.g. paediatricians) accessible to all clinicians
Equipment	Balance-beam or electronic scales (measure to nearest 0.1 kg)
	Fixed or correctly placed tape stadiometer (measure to nearest mm)
	BMI calculator (age and gender specific)
	Visual acuity (VA) chart suitable for pre-school children
	VA chart correctly placed (according to chart-type, 3 m or 6 m)
Examination method	Uses standardised developmental screening tool (e.g. PEDS^a^) as part of HKC
	BMI calculation and interpretation
	Tests uni-ocular vision (patches or covers the eye adequately)
	Applies “Lift-the-Lip” tool correctly

^a^PEDS = Parents’ Evaluation of Developmental Status

The HKC procedure was as follows: On arrival, office staff handed a PEDS questionnaire to the parent, to complete in the waiting room. This was scored by the practice nurse at the outset of the HKC-consultation so that the HKC was tailored to parent concerns. Body Mass Index (BMI) was calculated and interpreted following accurate measurement of height and weight. Uni-ocular visual acuity was assessed, and corneal light reflection and cover tests examined for strabismus. Oral health was assessed using the “Lift the Lip” tool [21]. Other mandatory components of the HKC were completed by direct questioning (Table 1). Where possible, parent concerns were addressed using parent tip-sheet resources. The business model proposed that each HKC, including PEDS interpretation, would be signed off by a GP, so that the parent could claim a Medicare rebate commensurate with time taken (and rebated more highly than the nurse-only HKC item). In the event that a developmental or health problem was discovered, practitioners could access additional resources provided in the electronic folder. A request was made for PEDS forms to be de-identified and returned to the research nurse to analyse concerns identified by parents.

### Measurements

A significant component of measurement utilised the Pen Computer System Clinical Audit Tool (PCS CAT) [22]. This tool has been widely implemented by PHOs to analyse practice population, Medicare and some clinical data. BMI is the only clinical output of the HKC recorded by the PCS CAT, but it is not exclusive to the HKC. Practitioners’ anthropometric measures, as part of routine child health care, would also be recorded by the PCS CAT. Practitioner questionnaires used 5-point Likert scales to assess beliefs and attitudes to prevention (11 questions) and self-efficacy (9 questions) across all age groups, including child health items about HKCs, developmental assessment and autism screening (Table 3). Questionnaires were tested for face validity. Training modules were also evaluated using brief questionnaires (Table 4). Practice inventories, adapted from surveys used in US-based research [23], recorded equipment and processes used by the practice during HKCs.

**Table 3:** Questionnaire and frequency distribution of responses

Questions asked of Clinicians (N = 14)		Before HKC- intervention					After HKC-intervention				
For child preventive health:-		Strongly disagree	Disagree	No opinion	Agree	Strongly agree	Strongly disagree	Disagree	No opinion	Agree	Strongly agree
Questions 1–6 = ‘Beliefs’											
Questions 7–12 = ‘Self-efficacy’											
(Adult preventive health items not included)											
1	I believe Early Intervention services are important in improving outcomes for children and families	0	0	0	3	11	0	0	0	3	11
2	I play a significant role in providing advice about vaccination	0	0	0	4	10	0	0	1	3	10
3	Our practice plays a significant role in providing vaccination services	0	0	0	4	10	0	0	0	4	10
4	I think it is important to calculate a BMI for school aged children	0	0	1	9	4	0	0	1	9	4
5	I think it is important to calculate a BMI for children aged 2 to 5 years	0	1	3	6	4	0	0	4	6	^b^4
6	I believe pre-school children should have their development assessed in general practice at every opportunity	0	0	1	6	7	0	0	0	8	6
7	I feel confident in my ability to conduct post-natal checks of infants	0	3	0	5	6	0	1	2	3	8
8	I feel confident in my ability to perform a Healthy Kids Check for a child aged 4.5 years	0	0	1	^a^9	4	0	0	1	6	7
9	I feel confident in my ability to perform a Healthy Kids Check for a child aged 3.5 years	0	1	1	8	4	0	0	3	4	7
10	I feel confident in my ability to detect developmental problems in pre-school children without the use of standardised developmental screening tests	1	1	5	5	2	0	2	4	6	2
11	I feel confident in my ability to use standardised developmental screening tests (e.g.PEDS) to help detect developmental problems in children < 5 years	0	0	6	7	1	0	0	2	9	3
12	I feel confident in my ability to detect the “red flags” for Autism in children under 5 years	0	1	4	7	2	0	1	3	7	3

^a^Missing data adjusted to reflect no change from data obtained in post-intervention questionnaire

^b^Missing data adjusted to reflect no change from data obtained in pre-intervention questionnaire

**Table 4:** Training questionnaire and frequency distribution of responses

Questions		Pre-workshop					Post-workshop				
How would you rate your….		Low				High	Low				High
	
1	Knowledge regarding how to access early intervention services for young children	1	5	7	3	1	0	0	2	6	9
2	Knowledge about which children are eligible for a Healthy Kids Checks	0	4	4	8	1	0	0	0	6	11
3	Knowledge about the item numbers associated with providing a Healthy Kids Check	2	4	1	6	4	1	1	2	4	9
4	Personal level of comfort asking parents to complete questionnaires about their child’s development	3	4	6	4	0	0	0	1	5	11
5	Knowledge of standardised developmental assessments like PEDS	5	7	2	3	0	0	1	0	6^a^	10

PEDS = Parents’ Evaluation of Developmental Status

^a^Missing data adjusted to reflect no change from data obtained in pre-intervention questionnaire

Baseline data included: The number of eligible-age children (aged between 3.5 and less than 5 years) as a proportion of the total “active” practice population (attended at least once in the previous 12 months), the number of HKCs completed in the previous 12 months, and the proportion of eligible children with a BMI recorded. Data, including numbers of age-eligible children and total practice population, were viewed sequentially using the PCS CAT on practice computers at 3 and 6 months following intervention. Practitioner questionnaires and practice inventories were recorded at baseline and on a second occasion between 3 and 6 months following intervention.

### Analysis

We calculated and compared the proportions of eligible children receiving a HKC and having a BMI recorded at baseline, 3 months and 6 months following intervention, using two proportion Z-tests. Median scores calculated from Likert scales of items testing practitioners’ beliefs, confidence and training, were compared before and after intervention using Wilcoxon signed rank tests, in SPSS [24]. Scores of practice inventories were also analysed before and after intervention. PEDS forms were analysed for numbers of predictive and non-predictive problems identified.

## Results

### Recruitment

Of the six practices that initially expressed an interest in the study, two later declined and one practice proved ineligible because it did not conduct HKCs. All three enrolled practices were privately owned clinics and provided services for children with no ‘out of pocket fees’ (accepted the Medicare rebate as the entire fee) (Table 5). Practice C was notable in serving large populations of recent migrants. The project nurse made six visits to each practice, with an additional five visits to one practice due to scheduling and data extraction problems.

**Table 5:** Population, billing type, ownership and clinicians servicing practices A, B and C

Practice descriptor	A	B	C
SEIFA^a^	981	1003	939
AEDI^b^ (%)	22.1	18.3	39.5
Practice population (baseline)	3950	9700	19750
Population eligible children (baseline)	575	1580	2600
Billing^c^	Mixed (some out-of-pocket fees)	Bulk billing only	Bulk billing only
Ownership	Privately owned	Privately owned	Privately owned
GPs	4	4	6
Practice Nurse	1	1	3

^a^Socio-economic Index for Areas (SEIFA) has a national average of 1000 with increasing disadvantage as values decrease. SEIFA is a suite of four indexes that have been created from social and economic Census information. Each index ranks geographic areas across Australia in terms of their relative socio-economic advantage and disadvantage [10]

^b^AEDI = Australian Early Developmental Index: Developmentally vulnerable on 1 or more domains- Victorian average 19.5 % [11]

^c‘^Bulk Billing’ No out-of-pocket fees for the patient. All practices bulk billed HKCs

### Participation

One practice nurse in each practice agreed to ‘champion’ the delivery of the HKC. Twenty of 22 practitioners (GPs and PNs) and practice managers attended a component of training (participation rate 91 %) but, due to time constraints, only 50 % GPs received the entire module. Ten GPs, 4 PNs, and 3 practice managers completed pre-post questionnaires. Reception-staff were invited to join the study (module 1) but were not requested to complete questionnaires.

### Training

For those clinicians and staff that completed training evaluation (n = 17), knowledge about the administration of HKCs and PEDS screening and personal comfort associated with requesting parents to complete PEDS, increased [5 items. *Mdn* 49, vs *Mdn* 76*, (Z =* 2.02*, p* .043*, r = .*90*)*] (Table 4).

### Practitioner questionnaires

At baseline, all practitioners held positive beliefs about the value of developmental assessment and early-intervention services and believed they played a significant role in adult preventive health. GPs and PNs were generally confident administering health assessments across all age groups, but three of the four PNs lacked confidence performing infant health checks, and one GP was less confident performing HKCs on younger children (<3.5 years) (Table 3).

Following intervention, 15 participants (94 %) believed the HKC- intervention had developed their skills working with young children, and all agreed that their practice had successfully implemented standardised HKC services. Overall, whilst practitioners maintained beliefs about child preventive healthcare [*Mdn* 64.0, vs *Mdn* 63.5, *(Z =* 0.82, *p .*414*, r* = .41)], confidence in ability to perform HKCs and developmental assessments increased post-intervention [*Mdn* 54.0, vs *Mdn* 58.5, (*Z* = 2.23, *p* .026, *r* = .91)].

Practitioners thought that it was important to calculate a BMI for adult patients and older children, however five practitioners (4 GPs and 1 PN) remained ambivalent about calculating BMI for children aged 2 to 5 years post-intervention. Post baseline questionnaires indicated that PNs rated either their role in the practice decreased in respect of another aspect of preventive health care (vaccination service delivery, screening for adult hypertension) or they felt less confident performing adult health checks. Our small sample size precluded subgroup analysis.

**Fig. 1:**
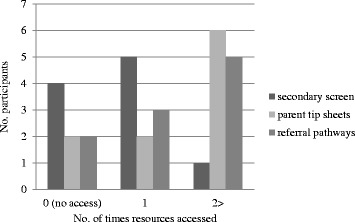
Use of desktop resources, secondary developmental screens, parent tip sheets and referral pathways, following HKC-intervention

Participants were asked if they had accessed their desktop ‘Community Resource Folder’. Ten practitioners responded that they had accessed parent tip-sheets, secondary developmental screens or referral pathways on one or more occasion (Fig. 1).

### HKC uptake

**Fig. 2:**
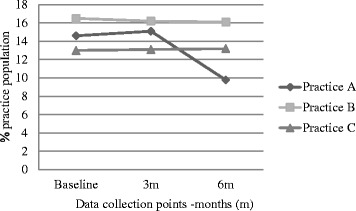
Population (%) of children eligible for HKC as proportion of practice population

**Fig. 3:**
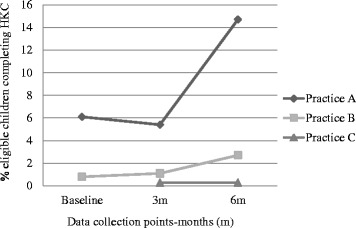
Proportion (%) of eligible children completing a HKC

**Fig. 4:**
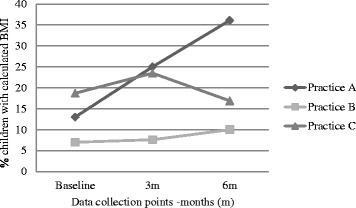
Proportion (%) of eligible children in each practice with calculated BMI

Over the 6 months, the proportion of eligible children within the practice population did not significantly change until the 6 month data collection point: for practice A, the overall population declined to 82.6 % baseline and the proportion of eligible children appeared to fall (Table 6 and Fig. 2). Practice A completed 22, practice B, 34 and practice C, at least 15 HKCs. The proportion of children completing a HKC significantly increased in two of the practices over the course of the study (Fig. 3). Due to software incompatibilities we were unable to obtain baseline HKC numbers, so could not calculate a baseline proportion for practice C. The proportion of eligible children who had a BMI recorded also significantly increased in practice A and B, but appeared to decrease in Practice C (Fig. 4).

**Table 6:** Proportions of eligible children completing HKCs and having BMI recorded

Parameter	Practice	Baseline (percent)	6 months after intervention (percent)	Z score	*P* value
Population of eligible children as proportion of practice population	A	14.6	9.8	6.13	0.
	B	16.5	16.1	0.77	.44
	C	13.0	13.2	−0.64	.52
Proportion of eligible children completing a HKC	A	6.1	14.7	−4.29	0.
	B	0.8	2.7	−3.9	0.0001
	C	-	-	-	-
Proportion of eligible children with BMI recorded	A	13.0	36.1	−8.06	0.
	B	7.0	10.0	−2.8	.005
	C	18.7	16.9	1.63	.10

### Inventories-Quality improvements

**Fig. 5:**
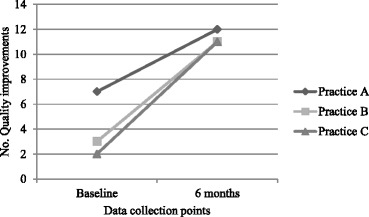
Quality Improvements in practice A, B and C following HKC intervention

All practices improved their equipment and processes of HKC-delivery. A total of 5 improvements to office systems, 7 equipment improvements and 10 improvements in examination methods (22 out of a total possible 24 improvements) across the three practices were made (Fig. 5) as detailed below:

i) Office systems
  All practices stated they had an accessible list of paediatricians, allied health practitioners, early intervention and community services in place before the study. Although all three practices used recall systems for adult health checks, only one practice invited children for HKCs. Remaining practices trialled recall systems by study conclusion. Under guidance from the intervention, all practices implemented procedures to handout PEDS from the reception area with each HKC.
ii) Equipment
  Access to measuring devices improved following implementation: One HKC- Champion ensured she used digital (rather than mechanical) scales and one practice corrected the placement of a tape stadiometer. A second practice was made aware of incorrect placement but had not re-placed it at study conclusion. HKC-Champions did not access *paediatric* BMI calculators until after the intervention (see below). Visual acuity testing required a valid eye chart (appropriate for testing the target age group) to be placed at the correct distance from the subject. By study conclusion one practice had corrected chart-type and placement, but a second practice continued to use a chart without a scale.
iii) Examination methods
  At baseline, although PCS CAT data implied that a proportion of children had a documented BMI, this proved to be an automated calculation based on adult categories of overweight. Direct questioning confirmed that BMI was not calculated or interpreted in the pre-intervention period by any of the HKC-Champions. At baseline, visual acuity was correctly assessed (uni-ocular) in only one practice and methods were adjusted by the intervention for the other two clinics. None of the practices utilised developmental screening tools before intervention and two out of three HKC-Champions did not use oral assessment tools until after intervention.

### PEDS questionnaires

Twenty-seven de-identified PEDS forms were returned for further analysis (6 from practice A, 13 from practice B and 8 from practice C). These identified 15 concerns, 6 of which were predictive of at least a moderate risk of disability (8.5 % sample). We were not able to determine the clinical decisions actually made for these children.

## Discussion

In Australia, all parents must produce an ‘Immunisation History Statement’ before their child is enrolled in school [25] and parents receiving income support must also obtain a health check for children turning four years of age [26], thus presenting opportunities for general practice to identify young children at risk and intervene to reduce disparities. Our study confirmed the feasibility of delivering a multi-faceted intervention to increase HKCs in general practice. In the US multi-faceted preventive child health interventions have been assembled as combinations of single element interventions, often without a clear rational for their choice [13]. Grimshaw et al. observed that an increase in dose of “component interventions” did not always lead to an increased response and proposed that multi-faceted interventions should be “built upon a careful assessment of barriers and a coherent theoretical base” [27]. In our study the elements that constituted the HKC-intervention were determined using a theoretically based behavioural change system. This pilot study demonstrated that the assembled package of intervention-components successfully incorporated solutions to the barriers identified in our primary research. Findings suggest that by upskilling the practice nurse and by taking a team approach, GPs were able to streamline processes, incorporate evidence-based preventive health care, standardise and improve quality and increase self-efficacy, delivering HKCs. The duration of the study was not long enough to determine if proportions of children completing HKCs in these practices ever attained the state-wide average of approximately 22 % [15]. An aspect of the intervention that worked less well was the training module for clinicians. Despite a flexible approach, the research team noted that GP-attendance was frequently interrupted by clinical demands so that training was incomplete for approximately half of attendees. ‘E-learning’ provides a flexible training method for clinicians [28] and has been successfully applied to paediatrics [29], presenting a potential solution in future trials of the intervention.

A second problem related to difficulties collecting data. Software changes for practice C, in the year before the study, precluded collection of baseline data. In addition, practice A undertook ‘database cleansing’ during the study, which produced an apparent large decline in total and age-eligible populations. General practices in Australia do not have fixed lists of patients, so that when practices decide to update patient databases they must determine which patients still ‘actively attend’. The commonly accepted definition of an ‘active’ patient, ‘attending three or more times in the past two years’ [30] differed from the less conservative definition employed in this study - ‘any patient attending at least once in the last 12 months’. This definition was decided upon as families access healthcare on behalf of their children from a variety of sources, and may not attend one practice on three occasions over 2 years. This may partially explain the extremely low proportions of children we recorded completing HKCs. In practice A, changes in the way patients were recorded over the course of the study may have artificially inflated the proportions of children documenting BMI and HKC improvements, although analysis at 3 months already showed improved HKC counts.

This study did not determine children’s health outcomes, or the referrals made as a result of HKCs, an additional barrier that influenced practitioner motivation in our previous research. A record was made of how many times practitioners thought they had accessed resources to address the outcomes of HKCs, however. Both ‘Parent tip sheets’ and ‘Referral pathways’ were accessed, suggesting that a significant proportion of problems were managed in-house. Secondary screens were accessed by a total of five GPs but only one PN. This implies that within the HKC process there is a degree of role separation. GPs are more likely to assume responsibility for decision making when problems are identified within a HKC, a practice reinforced in our business model, PEDS interpretation and training.

This pilot project demonstrated significant changes in measures of HKC uptake and BMI. However, the before and after study design means that we cannot be certain that our intervention was the sole reason for the observed differences. Anecdotally, staff informed the project nurse that a software upgrade, installed midway through the project in all practices, automated and correctly categorised BMI for children undergoing same day readings of height and weight. This automated measure would have enabled PNs to interpret readings and could account for the improvements in proportions of children having BMI recorded outside of HKCs, during sick-child consultations. Discussions with HKC-Champions revealed that they were not calculating or interpreting BMI prior to the intervention, but did not elucidate whether other clinicians were doing so. Practitioners’ relative ambivalence towards measuring BMI for young children, which following intervention remained unchanged for some GPs, also suggests further education may be needed.

Results show that relatively large quality improvements were made across three different practice areas: office systems, equipment and examination techniques. The practice that started from the lowest base made the largest gains (practice C), but all practices improved across each domain.

### Limitations

This intervention study employed a 3 month active intervention period with an additional 3 months of follow-up. It is not known if practices continued to deliver HKCs using this format following the study. It would be interesting to know, for example, if practices continued to acquire PEDS questionnaires or if systems can be maintained during staff turn-over. This study recorded equipment and processes but did not assess how effectively PNs conducted HKC-examinations. From the PEDS forms that were returned, it was estimated that a small proportion of children had concerns predictive for developmental delay. There was no way to determine if these problems were acted upon by the medical team or the parents. Future studies could be designed to address such issues.

This study was conducted in an area that serves large numbers of young families with pockets of high socioeconomic disadvantage and child developmental vulnerability [15]. It would, therefore, be important to test this intervention across diverse populations, allow longer follow-up periods and include control sites to avoid bias, before recommending full uptake.

### Negative effects

It is possible that by concentrating on one area of preventive health care in general practice, another sector lost out. PNs’ responses noted diminished participation, or reduced confidence, in other aspects of preventive health. This appears to be a valid observation because it is unlikely that participants would recall their responses to pre-intervention questionnaires. In the US, different preventive services have been found to compete with each other for physicians’ time, as well as with acute care [31] and caution has already been expressed about the opportunity costs of preventive services in Australian general practice [32].

### Lessons learned and steps towards a cluster randomised controlled trial

A cluster randomised (phase III) trial would provide further evidence of the effect size of the intervention and would test generalisability to other populations. Recruitment methods (through PHOs) will be extended to practice-based research networks already affiliated with the research team. It would be important to test the intervention in another Australian jurisdiction because Victoria’s CFN services operate differently to other states and may impact on GP service delivery, with a control arm to increase the strength of the study (usual care, including HKCs conducted without the practice based intervention).

This pilot study demonstrated that the intervention was acceptable and feasible, and confirmed the selection of outcome measures (an increase in the proportion of eligible children receiving HKCs and having BMI recorded, and significant quality improvements to practice processes and equipment). Data collection methods will extend over 12 months and the commonly accepted term for “active” patients will be adopted to maintain consistency within the data [30]. Paper-based surveys of practitioners will test GP and PN self-reported knowledge and self-efficacy (adapted from another preventive health study [33]) and health care utilisation following HKC will be captured (from government Medicare insurance services) to obtain important data regarding health outcomes. Arising from the pilot study was a recommendation to develop a web based module to streamline delivery of components of the training.

## Conclusions

Healthy Kids Checks have the potential to identify disabilities, health and behavioural concerns at a significant juncture for children and their families. This pilot study provides the first indications that it is possible to increase preventive healthcare for young children by increasing numbers of HKCs. A cluster randomised controlled trial would provide more definitive evidence for a multifaceted intervention, particularly if study sites were located across different states, and included a mix of practices. It would need to incorporate an evaluation of other aspects of preventive health, given that possible negative effects were detected in this study and it could be improved by incorporating research into the clinical outcomes of HKCs.

## Abbreviations

BMI: Body mass index
CFHN: Child and family health nurses
GP: General practitioner
HKC: Healthy Kids Check
PCS CAT: Pen Computer System Clinical Audit Tool
PEDS: Parents’ Evaluation of Developmental Status
PHO: Primary healthcare organisation
PN: Practice nurse
US: United States
